# Exopolysaccharide produced by *Lactiplantibacillus plantarum* RO30 isolated from Romi cheese: characterization, antioxidant and burn healing activity

**DOI:** 10.1007/s11274-022-03439-6

**Published:** 2022-10-26

**Authors:** Eman A. Elmansy, Ebtsam M. Elkady, Mohsen S. Asker, Amr M. Abdou, Nagwa A. Abdallah, Shaimaa K. Amer

**Affiliations:** 1 Microbial Biotechnology Department, Institute of Biotechnology Research, National Research Centre, El-Tahreer Street, Dokki, Cairo, 12622, Egypt; 2 Department of Microbiology and Immunology, National Research Centre, El-Tahreer Street, Dokki, Cairo, 12622 Egypt; 3 Microbiology Department, Faculty of Science, Ain Shams University, Cairo, Egypt

**Keywords:** Antibiotics, Antimicrobial activity, Exopolysaccharide, Lactic acid bacteria, Probiotic

## Abstract

Microbial exopolysaccharides (EPSs) extracted from lactic acid bacteria (LAB) are generally recognized as safe. They have earned popularity in recent years because of their exceptional biological features. Therefore, the present study main focus was to study EPS-production from probiotic LAB and to investigate their antioxidant and burn wound healing efficacy. Seventeen LAB were isolated from different food samples. All of them showed EPS-producing abilities ranging from 1.75 ± 0.05 to 4.32 ± 0.12 g/l. RO30 isolate (from Romi cheese) was chosen, due to its ability to produce the highest EPS yield (4.23 ± 0.12 g/l). The 16S rDNA sequencing showed it belonged to the *Lactiplantibacillus plantarum* group and was further identified as *L. plantarum* RO30 with accession number OL757866. It displayed well in vitro probiotic properties. REPS was extracted and characterized. The existence of COO^−^, OH and amide groups corresponding to typical EPSs was confirmed via FTIR. It was constituted of glucuronic acid, mannose, glucose, and arabinose in a molar ratio of 2.2:0.1:0.5:0.1, respectively. The average molecular weight was 4.96 × 10^4^ g/mol. In vitro antioxidant assays showed that the REPS possesses a DPPH radical scavenging ability of 43.60% at 5 mg/ml, reducing power of 1.108 at 10 mg/ml, and iron chelation activity of 72.49% and 89.78% at 5 mg/ml and 10 mg/ml, respectively. The healing efficacy of REPS on burn wound models in albino Wistar rats showed that REPS at 0.5% (w/w) concentration stimulated the process of healing in burn areas. The results suggested that REPS might be useful as a burn wound healing agent.

## Introduction

Probiotics are beneficial microorganisms with extraordinary traits and confer health advantages to the host when ingested in appropriate amounts (Khare and Gaur 2020). They achieve their beneficial effects on the host through various mechanisms including their ability to ensure gut microbial stability (Gasbarrini et al. 2016) and their ability to reinforce the host immunity (Faghfoori et al. 2015). They can be isolated from both dairy and non-dairy sources including animal products, and fermented food products. Probiotic bacteria are characterized by their tolerance to bile salts, low pH, and the effect of gastric juices. They have also the ability to combat detrimental microbes through the synthesis of antimicrobial agents. Furthermore, they have the capability to adhere to and colonize mucosal niches (Wang et al. 2020).

Numerous probiotic strains especially from genera of *Lactiplantibacilli* and *Bifidobacteria* have been explored (Reuben et al. 2020). Due to their long relationship with fermented foods, LAB are generally recognized as safe (GRAS) and have widely been investigated as probiotics for a wide range of commercial applications (Ayyash et al. 2020; Habib et al. 2022). The most used probiotic *Lactiplantibacillus* spp. are *Lactiplantibacillus acidophilus* (*L. acidophilus*), *Lactiplantibacillus casei* (*L. casei*), and *Lactiplantibacillus plantarum* (*L. plantarum*) among others.

*Lactiplantibacillus plantarum* is a versatile lactic acid bacterium, that is encountered in a range of environmental niches including dairy, meat and many vegetable fermentation (De Vries et al. 2006). Not long ago, *L. plantarum* has been inquired in health-promoting products as some strains have shown propitious clinical results, such as regulating gastrointestinal function, lowering serum cholesterol, and reinforcing immunity (Zhao et al. 2021).

The potential of EPSs production by certain probiotics is a desirable and necessary functional attribute as it assists them in resisting stress, effective gut settlement, and in quorum sensing (Habib et al. 2022). Additionally, EPSs produced by probiotic LAB have been selected for a range of applications in a variety of fields like textiles, cosmetics, bioremediation, food, pharmaceutical, and therapeutic industries (Ayyash et al. 2020). In the food industry, these biopolymers can positively influence the characteristics of the products such as texture and sensory qualities (Liu et al. 2019). Moreover, they provide vital health benefits through their antimicrobial, anti-biofilm, antioxidant, anti-cancer, anti-diabetic, anti-ulcer, anti-inflammatory, anti-viral activities, cholesterol-lowering effects, immunomodulatory properties and prebiotic properties (Angelin and Kavitha 2020). For instance, EPSs have been identified as the inducers of programmed cell death and autophagy in cancer cell lines (Di et al. 2017). Moreover, the findings of Maeda et al. (2004) revealed that the EPS (kefiran) from *L. kefiranofaciens* significantly suppressed the increase of blood pressure and blood glucose levels, while Tang et al. (2017) demonstrated antioxidant activity of EPS from *L. delbrueckii* ssp. *Bulgaricus* SRFM-1.

These biopolymers are retrieved from microorganisms as tightly bound capsule capsular polysaccharides (CPs) or loosely connected slime layer in microorganisms or emitted into the bacterial surroundings as exopolysaccharides (EPSs). According to the chemical composition and biosynthesis mechanisms, the EPSs from LAB can be categorized into two distinct groups: homopolysaccharides (HoPS) and heteropolysaccharides (HePS) (Angelin and Kavitha 2020). The chemical structure of EPSs is well known to serve as the basis for their biological function. As a consequence, the structure elucidation of EPS is extremely essential for investigating its biological activity. However, the structure analysis is quite difficult owing to the diversity and complexity of EPSs, particularly the determination of their three-dimensional structure (Liu et al. 2019).

Burns are the most extensive forms of soft tissue injuries occasionally causing extensive and deep wounds and death. Burns can lead to severe mental and emotional distress, because of excessive scarring and skin contractures. World Health Organization estimates that every year there are about 265 thousand deaths caused by burns on humans (Yuniarti and Lukiswanto 2017). Treatment of burns has always been a difficult medical problem and many different methods have been employed locally to repair such injuries. Some probiotic species have been proven to strengthen the process of wound repair in the gastrointestinal tract in various in vitro and in vivo models (Lukic et al. 2017). The existence of *L. rhamnosus* GG and *L. gasseri*, for instance, is thought to hasten the healing of stomach ulcers in rats. Probiotics have been shown to assist in the healing of gastric wounds, so researchers have begun to investigate whether they might also aid in the healing of cutaneous wounds (Sultana et al. 2013; Zaghloul and Ibrahim 2022). At the same time, derivatives of probiotics such as polysaccharides may be more suitable candidates for the development of effective and non-toxic wound healing agents with stronger activities (Ammar et al. 2015) to overcome obstacles related to maintaining the viability of living cells. Also, it is well known that when wounding occurs, the short-term process of inflammation triggered by the emission of the inflammatory mediators and radical oxygen species by the macrophages mainly hinders and delays the process of wound healing. As a consequence, suppressing the production of reactive radicals using EPS with antioxidant potential is a crucial aspect of attracting fibroblasts to the location to begin the proliferative phase of the repair or wound healing process (Houghton et al. 2005; Tam et al. 2011). Therefore, this study aimed to isolate EPS-producing LAB, evaluate the probiotic potential of the selected isolate, produce new safe EPS with antioxidant and burn wound healing activity and characterize its structural properties using different methods.

## Methods

### Samples collection for isolation of LAB from different sources

Different dairy (milk, yoghurt, Romi cheese, old cheese and baramilli cheese) and non-dairy samples (pastirma, pickle, and fresh vegetables namely, carrot, radish, cucumber, red cabbage, and cauliflower) were collected from common markets in Egypt. All samples were transported directly to the laboratory and stored in the refrigerator till further analysis.

For the isolation of LAB from fresh and pickled vegetables, 25 g of fresh and pickled vegetables was broken and suspended in 225 ml 0.85% (w/v) sterile saline water and homogenized for 5 min with a vortex mixing. Afterwards, a volume of 100 μl of serial dilutions (10^−3^–10^−6^) were spread plates on de Man-Rogosa and Sharp Agar (MRS, pH 5.8 ± 0.2) for isolation of *Lactiplantibacillus* spp. All plates were incubated for 48 h at 37 °C. After incubation, colonies showing different appearance (such as color, shape and size) were selected and further purified by successive streaking using the same medium. The pure colonies were examined for their cell morphology, Gram staining, catalase and oxidase tests. Gram-positive, catalase and oxidase-negative bacterial isolates were re-suspended and maintained in the same medium containing 20% (v/v) glycerol at − 80 °C for further studies (Abid et al. 2018).

### Isolation of EPS

For the EPS extraction, a protocol of Hu et al. (2021) with minor modifications was used. The MRS medium with 5% sucrose was inoculated (1%, O.D_620nm_ = 1.25) with an overnight bacterial culture and incubated for 72 h at 37 °C. Bacterial cells were removed by centrifugation at 4 °C, 4000 rpm in a refrigerated centrifuge (SIGMA 3–18 KS), and for 20 min. Then, trichloroacetic acid (TCA) was added to the supernatant at a final concentration of 6% (w/v), and the precipitated proteins were removed by centrifugation (4000 rpm at 4 °C for 20 min). The clear supernatant was collected, neutralized with NaOH and the EPS was precipitated by adding three volumes of cold absolute ethanol and maintained overnight at 4 °C. The precipitate was recovered by centrifugation at 4000 rpm for 10 min at 4 °C. The precipitate was dissolved in ultrapure water and dialyzed for 2 days at 4 °C against the same solution (changed twice each day), using a dialysis membrane having a cut-off of 3.5 kDa. After dialysis, the EPS was re-precipitated with three volumes of cold ethanol, and then dried in the oven at 35 °C, and the dry weight was taken as the amount of EPS produced. The phenol–sulfuric acid method was used to determine the total carbohydrates (Dubois et al. 1956), and protein content was determined using bovine serum albumin as a standard according to Bradford (1976). The bacterial isolate that produced the highest amount of EPS was selected for further studies.

### Physiological and biochemical characterization of the isolate RO30

The selected bacterial isolate was further characterized taking into account the following biochemical tests: carbohydrate fermentation (glucose, lactose, and sucrose) and mobility test, growth at 25, 35, 40 and 45 °C in MRS broth, tolerance to NaCl by growth in MRS broth containing 4.5, and 6.5% NaCl, growth at different pH 3.5, 4.5, 5.5, 6.5, 7.5, and 8.5 in MRS broth.

### Phylogenetic identification of the isolate RO30

Genetic identification of the selected isolate was performed by amplifying the 16S rRNA gene of the isolate RO30 using universal primers (27F 5ʹ-AGAGTTTGATCCTGGCTCAG-3ʹ and 1492R 5ʹ-GGTTACCTTGTTACGACTT-3ʹ). The presence of specific PCR products was confirmed by agarose gel electrophoresis. The DNA sequence of the PCR product was carried out by Macrogen Sequencing Facilities (http://dna.macrogen.com, Seoul, Korea). Sequence results were aligned with the NCBI database using the BLAST algorithm. Accession number was received for selected LAB isolate by GenBank. The neighbor-joining method was used to construct phylogenetic tree (Saitou and Nei 1987). Bootstrapping analysis of 1000 replications was performed to estimate the confidence of tree topologies (Felsenstein 1985).

### Probiotic properties of the RO30 strain

#### Acid and bile tolerance

The method of Saif and Sakr (2020) was used to determine acid and bile salt tolerance of the selected strain. Briefly, overnight cultures of the test isolate were inoculated (1% v/v) in MRS broth previously adjusted to pH 3.0 or treated with a bile salt concentration of 0.3%. After incubating for 3 h at 37 °C, 1 ml of the culture medium was mixed with 9 ml of MRS broth and incubated for 24 h at 37 °C. Resistance was assessed in triplicates in terms of optical density measurement at 620 nm. The control comprised MRS broth adjusted to pH 7.0 ± 0.2. The percentage of resistance was calculated as:

$${\text{Resistance }}\left( \% \right) \, = {\text{ OD}}_{{\text{s}}} /{\text{OD}}_{{\text{c}}} \times {1}00.$$

#### Cell surface hydrophobicity

Bacterial cells from overnight culture were harvested, washed twice and re-suspended in 10 ml of phosphate-buffered saline (PBS) (pH 7.0). The initial absorbance (A_0_) of the suspension was adjusted with PBS to OD _620 nm_ = 1.0 (A_0_). One millliter of cell suspension in PBS buffer (A_0_) was dispensed in a clean and dry round bottom test tubes followed by the addition of an equal volume of *n*-hexane, xylene, and toluene. The contents were vortexed for 2 min. The tubes were left undisturbed for 1 h at 37 °C to allow phase separation. The lower aqueous phase was carefully removed with a sterile Pasteur pipette and absorbance (A_1_) was recorded (García-Hernández et al. 2016). Cell surface hydrophobicity in terms of percent (H%) was calculated using the following formula:

$${\text{Hydrophobicity }}\left( \% \right) \, = \, \left( {{1} - {\text{A}}_{{1}} /{\text{A}}_{0} } \right) \, \times { 1}00.$$

#### Antimicrobial potential

The antimicrobial potential of the RO30 strain was determined using the agar well diffusion method with some modifications of the protocol indicated by Mulaw et al. (2019) against *Bacillus cereus* ATCC 33018, *Salmonella typhimurium* ATCC 14028, *Escherichia coli* 0157: H7ATCC 6933, *Listeria monocytogenes* V7 strain serotype 1, *Staphylococcus aureus* ATCC 20231, *Pseudomonas aeruginosa* ATCC 9027 and *Candida albicans* as test pathogens. A volume of 50 µl grown test pathogen cells (~ 1.3 × 10^7^ CFU/ml) were seeded on nutrient agar plates. Uniform wells of a diameter of 7 mm each were cut on the dried agar plate using a sterile cork borer. Each well was filled with 100 µl of the cell-free supernatant, obtained by harvesting 24 h old culture of *L. plantarum* RO30 from MRS broth. The plates were first incubated at 4 °C for 60 min to allow the test material to diffuse in the agar and then incubated at 37 °C for 24 h. After incubation, the diameter of the clear zone around each well was measured in millimeters.

### Safety evaluation of the RO30 strain

#### Hemolytic activity

Fresh bacterial culture was streaked in triplicates on MRS agar plates, containing 5% (w/v) human blood, and incubated for 48 h at 37 °C. Blood agar plates were examined for signs of β-haemolysis, α-haemolysis or γ-haemolysis. Experiments were made at least three times on separate days (Saif and Sakr 2020).

#### Determination of antibiotic susceptibility

The susceptibility of the RO30 strain to different types of antibiotics was evaluated using the agar disc diffusion method (Bauer 1966). Commercially available antibiotics disc (Oxoid) including ciprofloxacin (5 μg), imipenem (10 μg), fusidic acid (10 μg), norfloxacin (10 μg), streptomycin (10 μg), aztreonam (30 μg), ofloxacin (5 μg), and clindamycin (2 μg) were used. Overnight culture was aseptically swabbed on MRS agar plates and the antibiotics discs were placed on the surface of the agar plates. The inoculated plates were incubated at 37 °C for 24 h and zone of inhibition were measured and recorded in millimeters.

### Physiochemical characterization of REPS

#### Monosaccharide analysis and molecular weight (MW) determination

The monosaccharide composition analysis of REPS was carried out using high-performance liquid chromatography (HPLC) (Adebayo-Tayo and Fashogbon 2020). The REPS sample (15 mg) was hydrolyzed using 5 ml of 85% formic acid at 105 °C for 5 h (Evans and Linker 1973), evaporate excess formic acid, wash several times with deionized water to remove the remaining formic acid, dissolve in absolute ethanol to collect mono sugars only, then evaporate the ethanol and dissolve the precipitate in deionized water and subsequently analyzed. The monosaccharide contents were quantified by HPLC on a Shimadzu Shim-Pack SCR-101N column (7.9 mm, 30 cm), using deionized water as the mobile phase (flow rate 0.5 ml/min), as described by El-Sayed et al. (2005).

High performance liquid chromatography/gel permeation chromatography (HPLC/GPC) system with refractive index 2414 detector equipped with Agilent PL-aquagel-OH-40 (300 × 7.5) column was used to determine REPS average molecular weight (Mw), average molecular number (Mn), and polydispersity index (PI). REPS solution was prepared by dissolving 50 mg of REPS sample in 10 ml of the diluent (100 mM NaCl, 10 mM NaH_2_PO_4_, 0.02% NaN_3_), and mixed thoroughly. The solution was sonicated for 5 min, filtered using 0.2 µ filter units and injected into the system and elution was carried out at a flow rate of 0.8 ml/min at 35 °C for 25 min. The standard solution was injected followed by sample solutions in duplicate.

#### FT-IR and ^1^HNMR spectroscopy

To identify the REPS functional groups, the dried REPS sample was mixed and ground with KBr crystals in ratios (1:100) and pressed into a disc. The FTIR spectrum was recorded on a Bruker Tensor 27 instrument with a resolution of 4 cm^−1^ in the region of 4000–400 cm^−1^ (Vidhyalakshmi et al. 2016).

To determine ^1^HNMR spectra, 50 mg of REPS was dissolved in 1 ml of mixture of Dimethyl sulfoxide and Trifluoroacetic anhydride and analyzed on a JEOL spectrometer operating at 500 MHz, and the chemical change was expressed in parts per million (ppm).

### Determination of the antioxidant potential of the REPS

#### The free radical scavenging activity of the REPS (DPPH)

The free radical scavenging activity of the EPS was measured by 2,2-diphenyl-2-picrylhydrazyl (DPPH) radicals according to Hu et al. (2021). Ascorbic acid was used as a positive control. The capability to scavenge the DPPH radical was calculated using the following equation:

$${\text{Scavenging activity }}\left( \% \right) \, = \left[ {1 - \left( {\frac{{\text{absorbance of sample}}}{{\text{absorbance of control}}}} \right) \times 100\% } \right].$$

#### The reducing power assay of the REPS

The reducing power of the different concentrations of REPS (4, 6, and 10 mg/ml) was evaluated using the method of Hu et al. (2021).

##### Metal ion chelating activity

The Fe^+2^ chelating ability of REPS was determined according to the method of Hu et al. (2021). The chelating antioxidant activity was calculated according to the following equation:

$${\text{Chelating activity }}\left( \% \right) \, = \left[ {1 - \left( {\frac{{\text{absorbance of sample}}}{{\text{absorbance of control}}}} \right) \times 100\% } \right].$$

### In vivo study of the effect of REPS on burn wound healing

#### Preparation of REPS sample

The crude REPS was dissolved at a concentration of 0.5% (w/w) in a sterile solution of stearic acid (12%), cetyl alcohol (1%), potassium hydroxide (1%), boric acid (0.8%), and glycerin (15%) and completed to 100 ml by distilled water with stirring until an ointment was obtained.

#### Burn healing properties of REPS

##### Animals

Five adult albino Wistar rats, weighing 185 ± 2.00 g were obtained and kept in the animal house unit of the National Research Centre, for at least 1 week prior to the experiments under standard conditions of light and temperature. All animals had access to standard laboratory diet consisting of vitamin mixture (1%), mineral mixture (4%), corn oil (10%), sucrose (20%), cellulose (0.2%), casein (10.5%), and starch (54.3%). Food and water were supplied ad libitum throughout the duration of the study.

##### Induction of burn wounds

The method of induction of second-degree thermal injuries, in the animals, was carried out according to Tavares Pereira et al. (2012) with minor modifications. In brief, first, the rats were anaesthetized with ether and the hair on the backside of the animals was removed. Then, the area was antisepticised with 1% povidone-iodine. A solid aluminum bar (diameter = 10 mm) was heated to a temperature of nearly 97 °C and pressed to the shaved and disinfected back of the animals for 20 s to induce three burning positions (diameter of each burn = 10 mm) on the back of each rat. The three burning positions were treated as follows: N = Negative control, the burn was treated daily with normal saline; C = Control, the burn was treated daily with the cream base as the vehicle only and (T = test group was treated daily with REPS mixed with the cream base at a concentration of 0.5%. All the treatments were applied, once a day, in a fine layer covering the surface of the burns using a sterile cotton swab. An analgesic was administered to all animals after the induction of burns to prevent animals from suffering. Then, each animal was placed in a separate cage until the end of the experiment to avoid licking or biting wound areas by other animals. On Days 2, 4, 6 and 7 after burning induction, color photographs of the wounds were taken by digital camera. On the last day (Day 7) of the experiment, animal termination was done by decapitation under anesthesia and their debris was disposed of according to the guidelines of the ethics committee of the National Research Centre.

##### Evaluation of burn healing ability of REPS

*Rate of burn wound closure* To evaluate the rate of burn healing in each group, the percentage of contraction of burn area in each group was calculated. A digital image of the burn area was taken on days 2, 4, 6, and 7 after the induction of burn using a digital camera placed at a fixed distance from the rats. The burn areas were then measured using Fiji image processing software and the percentage of burn contraction was calculated based on the equation (Barzegari et al. 2017),

$${\text{X}} = \, \left[ {\left( {{\text{A}}_{{2}} - {\text{A}}_{{\text{x}}} } \right)/{\text{A}}_{{2}} } \right] \, \times {1}00,$$

where A_2_ is the surface area of burn on the second day and A_x_ is x day.

##### Histopathological evaluation of burn healing ability of REPS

Autopsy samples were taken from the skin of rats on day 7 in burn areas with normal tissue surrounding them and fixed in 10% formal saline for 24 h. Washing was done in tap water then serial dilutions of alcohol (methyl, ethyl and absolute ethyl) were used for dehydration. Specimens were cleared in xylene and embedded in paraffin at 56 °C in a hot air oven for 24 h. Paraffin bees wax tissue blocks were prepared for sectioning at 4 microns thickness by LEITZ ROTARY microtome. The obtained tissue sections were collected on glass slides, deparaffinized, and stained by hematoxylin and eosin stain for routine examination through the light electric microscope (Bancroft and Gamble 2008).

### Statistics

All experiments were performed in triplicate, and mean values are presented. The results were given as mean ± standard deviation (SD). Statistical analyses were made using IBM SPSS Statistics 20.

## Results

### LAB isolation and purification

From the collected samples, a total of 62 bacterial isolates were obtained. Out of them, 17 isolates were found to exhibit negative catalase and oxidase activity and preliminarily identified as LAB.

### Screening of LAB isolates for EPS production

**Fig. 1:**
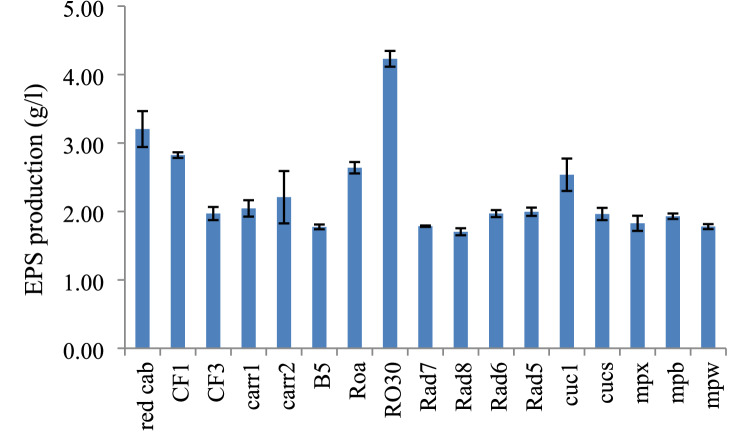
EPS production by different isolated LAB

A total of 17 LAB were estimated for their proficiency to manufacture EPS. Figure 1 clarified that isolate RO30 has the ability to create a maximum quantity of EPS (4.23 ± 0.12 g/l) in MRS medium supplemented with 5% sucrose. Other isolates exhibited a comparatively lower amount of EPS. The promising isolate was obtained from Romi cheese and was selected for further analysis.

### Morphological characters of potent LAB

**Fig. 2:**
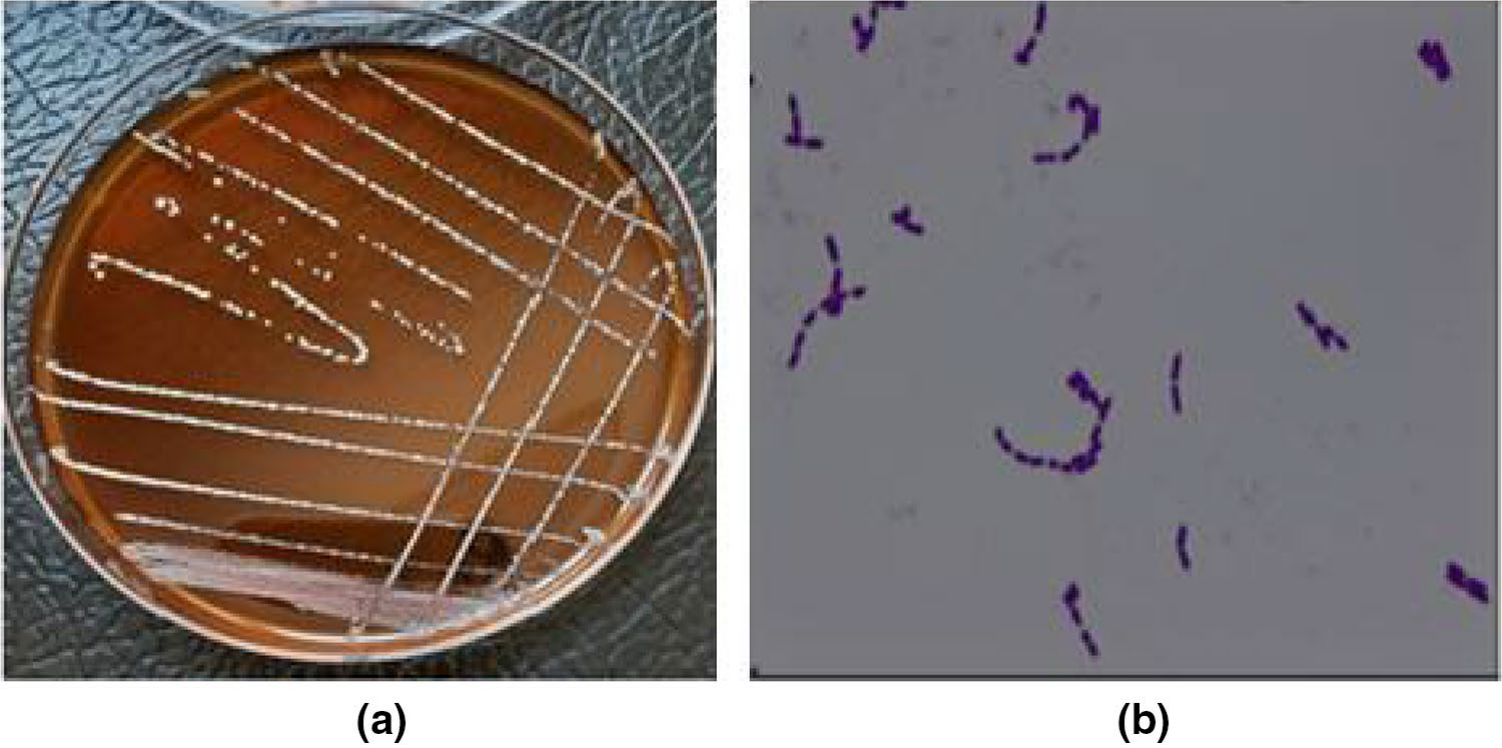
Culture characteristics (**a**) and microscopic examination (**b**) of isolate RO30

Taking into consideration the highest yield of EPS, isolate RO30 was selected and classified utilizing morphological, biochemical, and molecular approaches. RO30 colonies were smooth, small, circular, opaque, and creamy white (Fig. 2a). The isolate was gram-positive, rod-shaped (Fig. 2b), and non-spore-forming bacteria at × 100 magnification of the light microscope.

### Physiological characteristics of *L. plantarum* RO30

Concerning growth temperature, our *L. plantarum* RO30 grew well at 25, 35 and 40 °C, but a reduction in the growth was detected at 45 °C.

Regarding pH and salt concentrations, the obtained results revealed that *L. plantarum* RO30 not only tolerates different salt concentrations (4.5 and 6.5) but also tolerates wide pH ranges (4.5, 5.5, 6.5, 7.5 and pH 8.5) with ideal growth at pH 5.5.

Glucose fermentation test revealed that *L. plantarum* RO30 strain was homo fermentative bacteria i.e. produce only lactic acid as a primary by-product in glucose fermentation without gas production.

### Molecular identification of the promising bacterial isolate

**Fig. 3:**
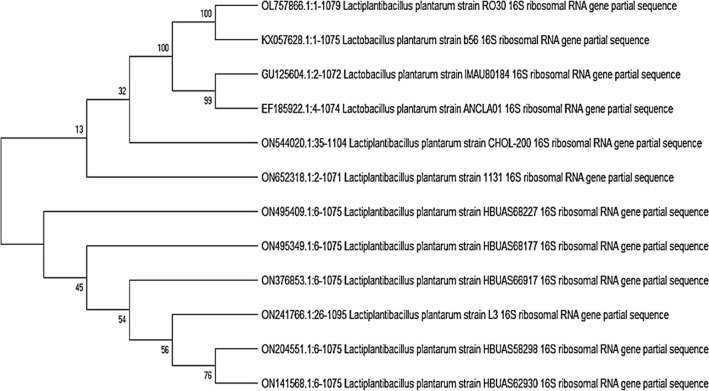
Phylogenetic tree of *L. plantarum* RO30

The total nucleotide sequence of 1079 bp was specified from the whole 16S rRNA gene of strain RO30 and kept in GenBank under accession number OL757866. The sequence alignment with preceding 16S rRNA gene sequences provided in the Genebank database revealed high closeness to the *Lactiplantibacillus* 16SrRNA reference genes thereby being classified as *Lactiplantibacillus plantarum* RO30. The phylogenetic tree was constructed using neighbor-joining tree method with the bootstrap method using the software MEGA10 (Fig. 3).

### Evaluation of probiotic characteristics of *L. plantarum* RO30

#### Acid and bile salt tolerance

In the present study, *L. plantarum* RO30 was challenged at low pH and it showed a survival rate of 98.63 ± 0.72% after 3 h.

Concerning bile salt tolerance, *L. plantarum* RO30 showed considerable resistance to exposure to 0.3% bile salts and a survival rate of 93.96 ± 0.56% was recorded.

#### Hydrophobicity assay

The microbial partition into different solvents as n-hexane, xylene, and toluene was utilized to assess cell surface hydrophobicity of *L. plantarum* RO30. The hydrophobicity of *L. plantarum* RO30 was found to be 19.54 ± 0.24, 34.61 ± 0.53, and 16.10 ± 0.35%, respectively.

#### Antimicrobial potential of *L. plantarum* RO30

**Fig. 4:**
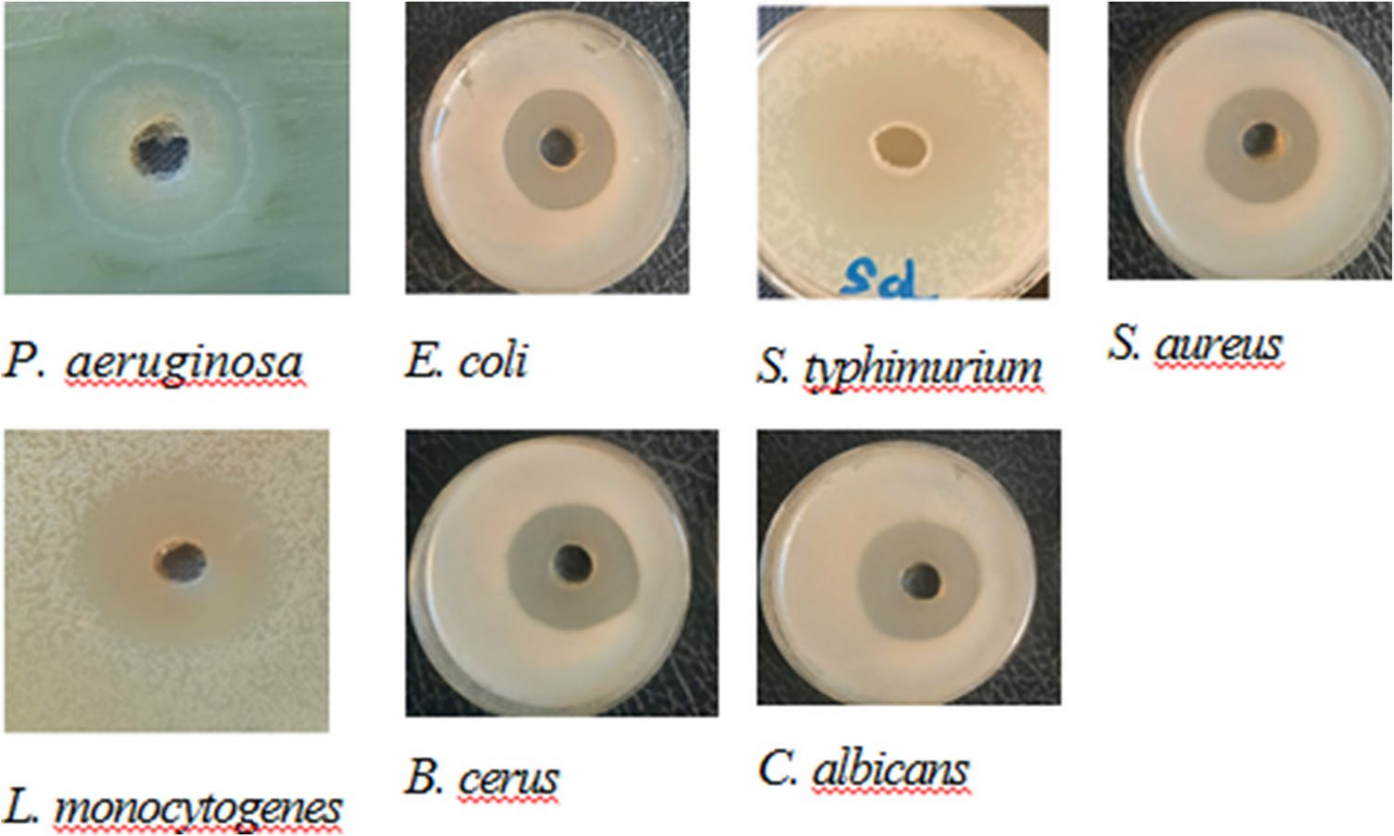
The antimicrobial activity of *L. plantarum* RO30 cell free supernatant

Screening of the antimicrobial activity of *L. plantarum* RO30 was assessed and the CFS displayed varying zones of inhibition with *monocytogenes* V7 strain serotype 1 (17.0 mm), *S. typhimurium* ATCC 14028 (14.5 mm), *S. aureus* ATCC20231 (8.5 mm), *E. coli* 0157: H7ATCC 6933 (15.0 mm), *B. cereus* ATCC 33018 (14.5 mm), *P. aeruginosa* ATCC 9027 (15.0 mm), and *C. albicans* (18.5 mm), thus revealing different responses by pathogens (Fig. 4).

#### Safety evaluation of *L. plantarum* RO30

##### Blood hemolysis

*L. plantarum* RO30 did not manifest any hemolysis on the agar blood plates (γ-hemolytic).

##### Antibiotic resistance of *L. plantarum* RO30

Data in Table 1 revealed that *L. plantarum* RO30 had variable susceptibility (high to low) towards penicillin, imipenem, chloramphenicol, tetracycline, clindamycin, gentamicin, ciprofloxacin, and ofloxacin. On the other hand, it was resistant to aztreonam, norfloxacin, and streptomycin.

**Table 1:** The antibiotic susceptibility of *L. plantarum* RO30

Class	Antibiotic	Concentration	Inhibition zone diameter (mm)	Sensitivity
Aminoglycoside	Streptomycin	10 µg	–	Resistant
	Gentamicin	10 µg	4.4	Low
Tetracyclines	Tetracycline	30 µg	11.5	intermediate
Amphenicols	Chloramphenicol	30 µg	15.0	High
Β-Lactams	Penicillin	10 IU	20.0	High
	Aztreonam	30 µg	–	Resistant
	Imipenem	10 µg	20.0	High
Quinolones	Ciprofloxacin	5 µg	4.4	Low
	Norfloxacin	10 µg	–	Resistant
	Ofloxacin	5 µg	3.5	Low
Lincosamides	Clindamycin	2 µg	5.0	Low

### Physiochemical characterization of REPS

#### Monosaccharide composition and MW determination of REPS

REPS produced by *L. plantarum* RO30 showed total carbohydrate content of 85.83% (w/w), protein content of 3.93% (w/w), and the moisture and ash was about 10.24% (w/w), indicating that the main component was polysaccharide.

The results of the REPS monosaccharide composition strongly indicated that the REPS is a heteropolysaccharide with glucuronic acid, mannose, glucose, and arabinose as monomer units with a molar ratio of 2.19:0.1:0.536:0.104, respectively.

GPC estimated the molecular weight using a calibration curve of the elution retention time of standard. REPS had a single major fraction with a Mw of 4.96 × 10^4^, Mn of 3.24 × 10^4^ g/mol, and PI of 1.53.

#### FT-IR and ^1^HNMR spectroscopy of REPS

**Fig. 5:**
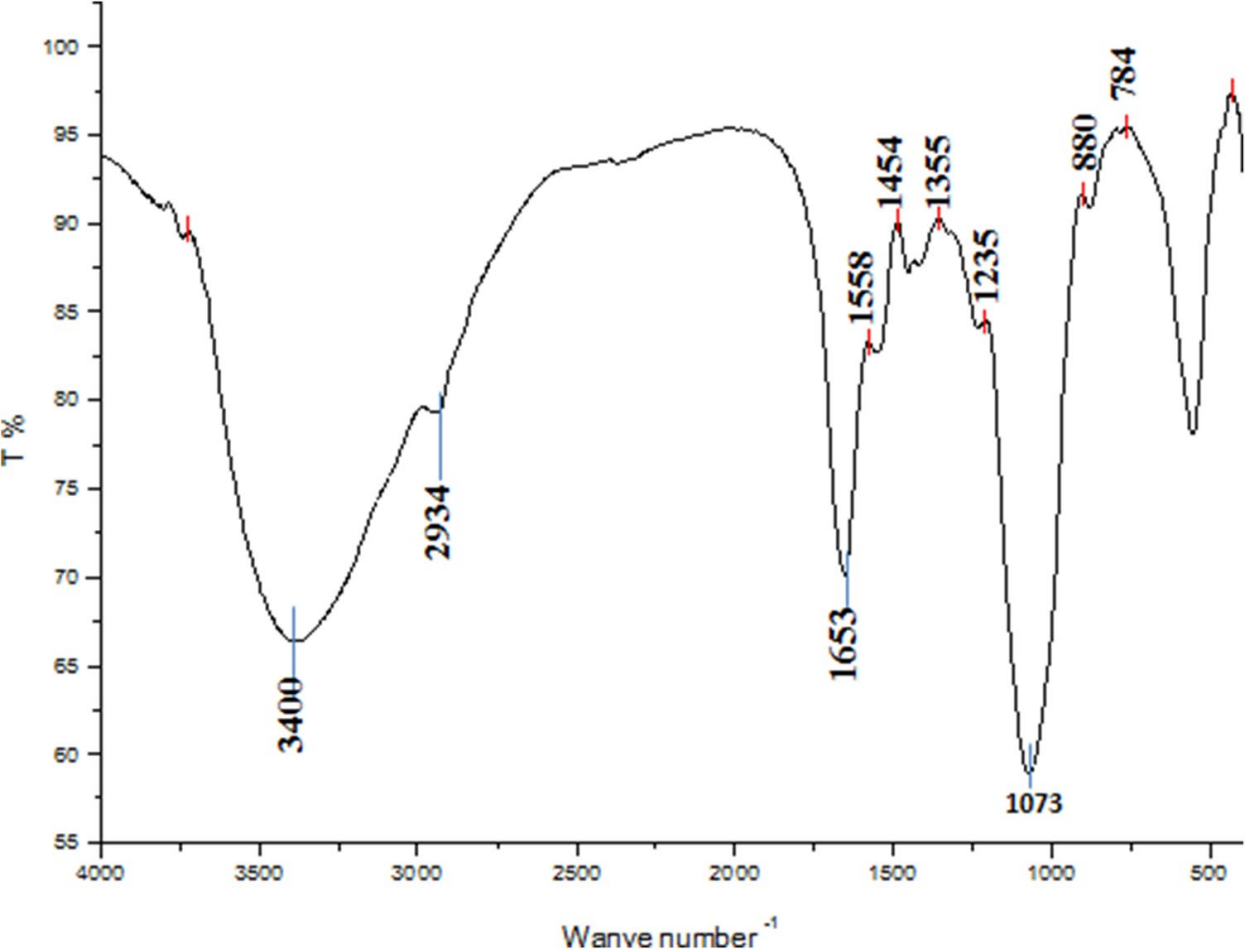
FT-IR of REPS from *L. plantarum* RO30

The FT-IR spectrum of the functional groups present in REPS identified plentiful peaks within the 4000–400 cm^−1^ range proving its polymeric structure (Fig. 5). The REPS exhibited absorption peaks at around 3400 cm^−1^, 2934 cm^−1^. Also, an intense extending peak at around 1653 cm^−1^ was observed. The peaks at around 1558 cm^−1^, 1454 cm^−1^, 1418 cm^−1^, 1235 cm^−1^, 1073 cm^−1^, 880 cm^−1^, and 784 cm^−1^ were also obtained.

**Fig. 6:**
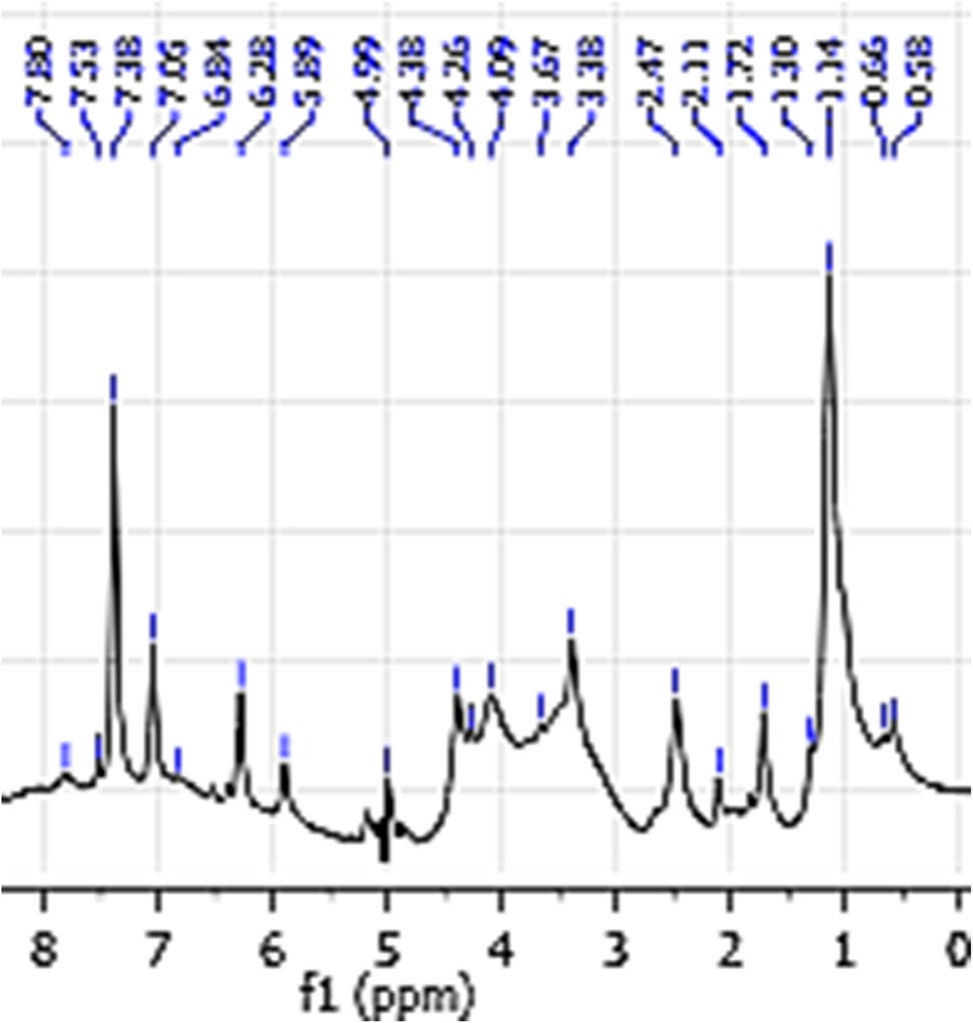
^1^HNMR spectroscopy analysis of REPS

The results of the ^1^HNMR spectrum showed that anomeric signals at *δ* 5.89, 4.99, 4.39, 4.09, 3.38 and 3.67 ppm were detected for REPS (Fig. 6).

### Anti-oxidant activity of REPS

#### The free radical scavenging activity (DPPH)

At 5 mg/ml concentration REPS expressed 43.60% DPPH radical-scavenging activity. The scavenging activity of REPS was lower than that of ascorbic acid (Vc) about 56.0%.

#### The reducing power assay of the REPS

The obtained results of Fe (III) ion reduction showed that the reductive activity of REPS using the K_3_Fe(CN)_6_ reduction method increased with increasing concentration. REPS at a higher concentration demonstrated a maximum reducing power ability of 1.1081 in terms of absorbance at 10 mg/ml REPS concentration, indicating that it has reductive potential.

#### Metal ion chelating activity

REPS showed an effective capacity for iron (II) binding and chelated almost 72.49% and 89.78% at 5 mg/ml and 10 mg/ml REPS concentration, respectively.

### Burn healing properties of REPS

#### Rate of burn wound closure

**Fig. 7:**
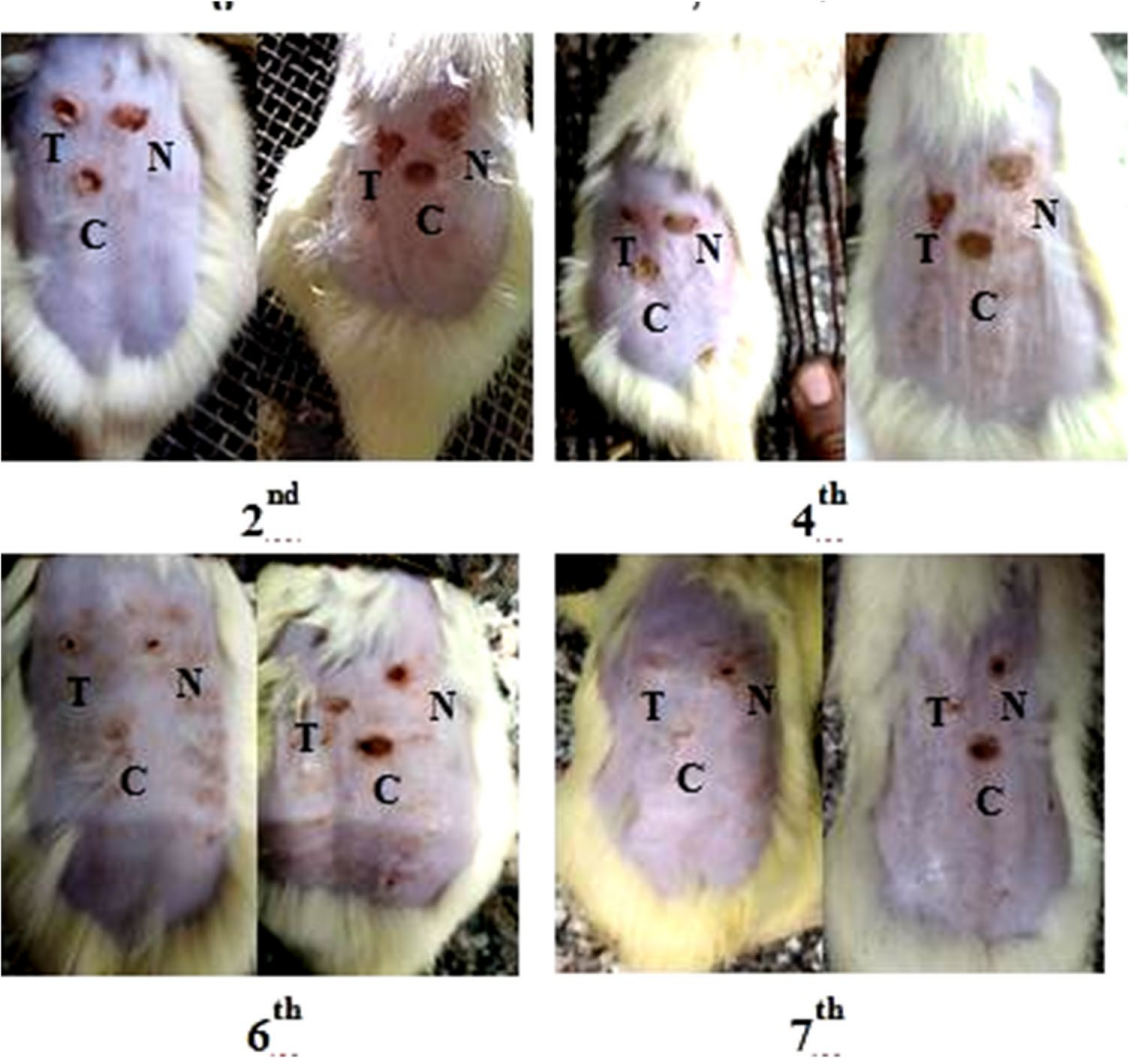
Burn induction in rats showing the healing process of burn areas of two representative rats at different time intervals. Where, *N* negative control, the burn was treated daily with normal saline, *C* control, the burn was treated daily with the cream base only (reference group), *T* test group was treated daily with REPS mixed with cream base at concentration 0.5%

To assess the degree of burn healing in rats, color images of lesions were obtained using a digital camera on days 2, 4, 6, and 7 after burn induction using a digital camera (Fig. 7). Figure 7 showed that REPS accelerated the process of healing of burn area when compared with the burn areas which were either treated with normal saline (N) or those treated with the cream base only (C).

Also, the size of the wound area was monitored during the 7-days experimental period to assess the burn wound healing potential of REPS. Results of wound closure are shown in Table 2. Significant wound healing activities were observed in rats treated with the cream base containing 0.5% REPS compared with the control and negative groups. Wound closure percentage in the rats treated with REPS was recorded as 64.88 ± 4.29% on the 4th day and 87.16 ± 11.30% on the 7th day. While in normal saline-treated rats, we observed 41.06 ± 16.78% and 65.34 ± 10.41%, respectively. In cream base treated rats, burn wound closure percentage was 45.52 ± 16.85% and 69.46 ± 6.86% on the 4th day and 7th day, respectively. Afterwards, there were no significant differences between the negative normal saline-treated control group and the control group that was treated with cream base only.

**Table 2:** Percentage of burn area contraction

Rat no.	Burn healing percentage (%)								
	Test group (T)			Negative control group (N)			Control group (C)		
	Day 4	Day 6	Day 7	Day 4	Day 6	Day 7	Day 4	Day 6	Day 7
1	60.1	62.4	79.3	50.0	55.0	59.1	28.8	51.5	62.4
2	63.0	74.2	100.0	22.9	34.6	54.2	28.1	41.6	64.8
3	64.2	69.3	73.9	62.5	68.1	80.0	58.3	60.7	66.6
4	71.7	82.2	85.2	25.4	49.2	61.6	65.2	70.8	75.7
5	65.4	86.2	97.4	44.5	62.3	71.8	47.2	65.6	77.8
Mean ± SD	64.88 ± 4.29	74.68 ± 9.61	87.16 ± 11.30	41.06 ± 16.78	53.84 ± 12.92	65.34 ± 10.41	45.52 ± 16.85	58.04 ± 11.62	69.46 ± 6.86

#### Histopathological evaluation

**Fig. 8:**
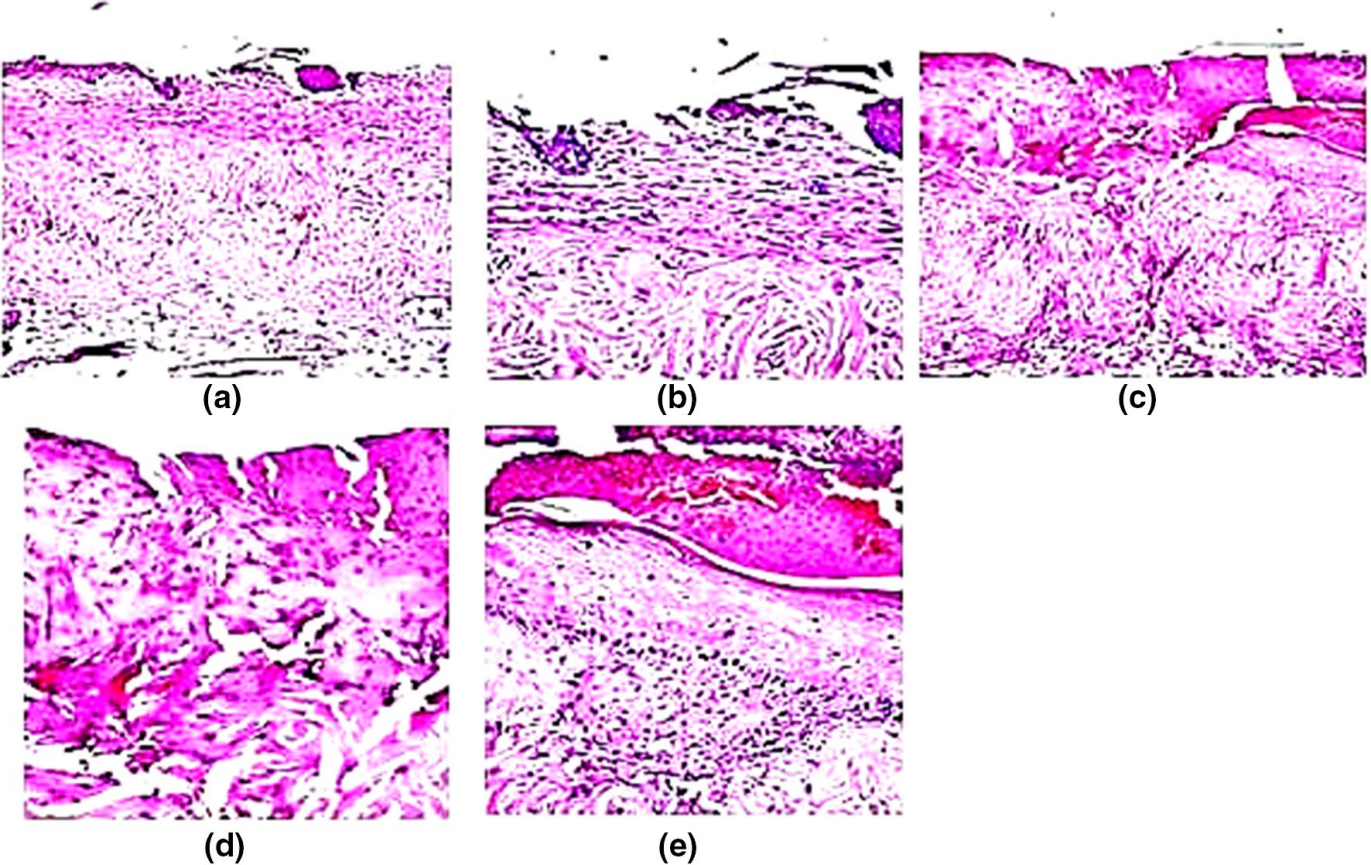
**a** Skin of rat showing focal ulceration in epidermis with underlying hyalinization, few inflammatory cells infiltration and few fibroblastic cells proliferation. **b** Showing the magnification of a to identify the focal ulceration in epidermis (H&E, × 40). **c** Showing focal ulceration and necrosis in the epidermis and underlying dermis with scale formation in adjacent area with acanthosis and few inflammatory cells infiltration. **d** Showing the magnification of **c** to identify the focal ulceration and necrosis in the epidermis and dermis (H&E, × 40). **e** Showing the magnification of **c** to identify the scale overneath the acanthotic epidermis and few inflammatory cells underlying the dermis (H&E, × 40)

To evaluate the degree of burn wound healing, hematoxylin–eosin stained rat skin tissue of negative control (normal saline), control (cream base only), and REPS treated groups were conducted at the final of the experiment. Histopathological examination confirmed the visual observations of the burn healing process. In the group of mice which were treated with normal saline (N), focal ulceration was observed in the epidermis associated with hyalinization and few inflammatory cells infiltration as well as few fibroblastic cells proliferation in the underlying dermis (Fig. 8a, b). In the same group, focal ulceration and necrosis were detected in the epidermis while the adjacent area showed acanthosis and over scale formation in the epidermis and few inflammatory cells infiltration in the dermis (Fig. 8c–e).

**Fig. 9:**
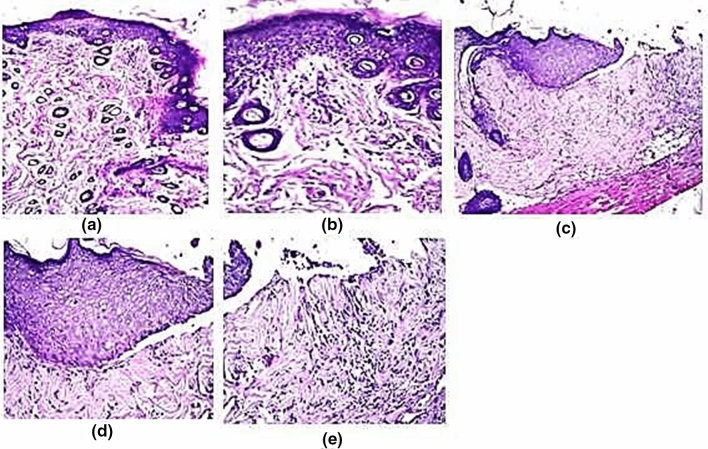
**a** Showing hyperkeratosis and acanthosis with damage hair follicles in the epidermis with underlying edema and few inflammatory cells infiltration in the underlying dermis. **b** Showing the magnification of **a** (H&E, × 40). **c** Showing focal acanthosis in the epidermis with adjacent ulceration and inflammatory cells infiltration in the dermis. **d** Showing the magnification of **c** to identify the focal acanthosis in the epidermis (H&E, × 40). **e** Showing the magnification of **c** to identify the focal ulceration in the epidermis with inflammatory cells infiltration in the underlying dermis (H&E, × 40)

In (C) group which was treated with cream base only, the epidermis showed hyperkeratosis and acanthosis with damage hair follicles while the underlying dermis had edema with few inflammatory cells infiltration (Fig. 9a, b). There was also focal acanthosis in the epidermis associated with adjacent area of ulceration with underlying inflammatory cells infiltration in the dermis (Fig. 9c–e).

**Fig. 10:**
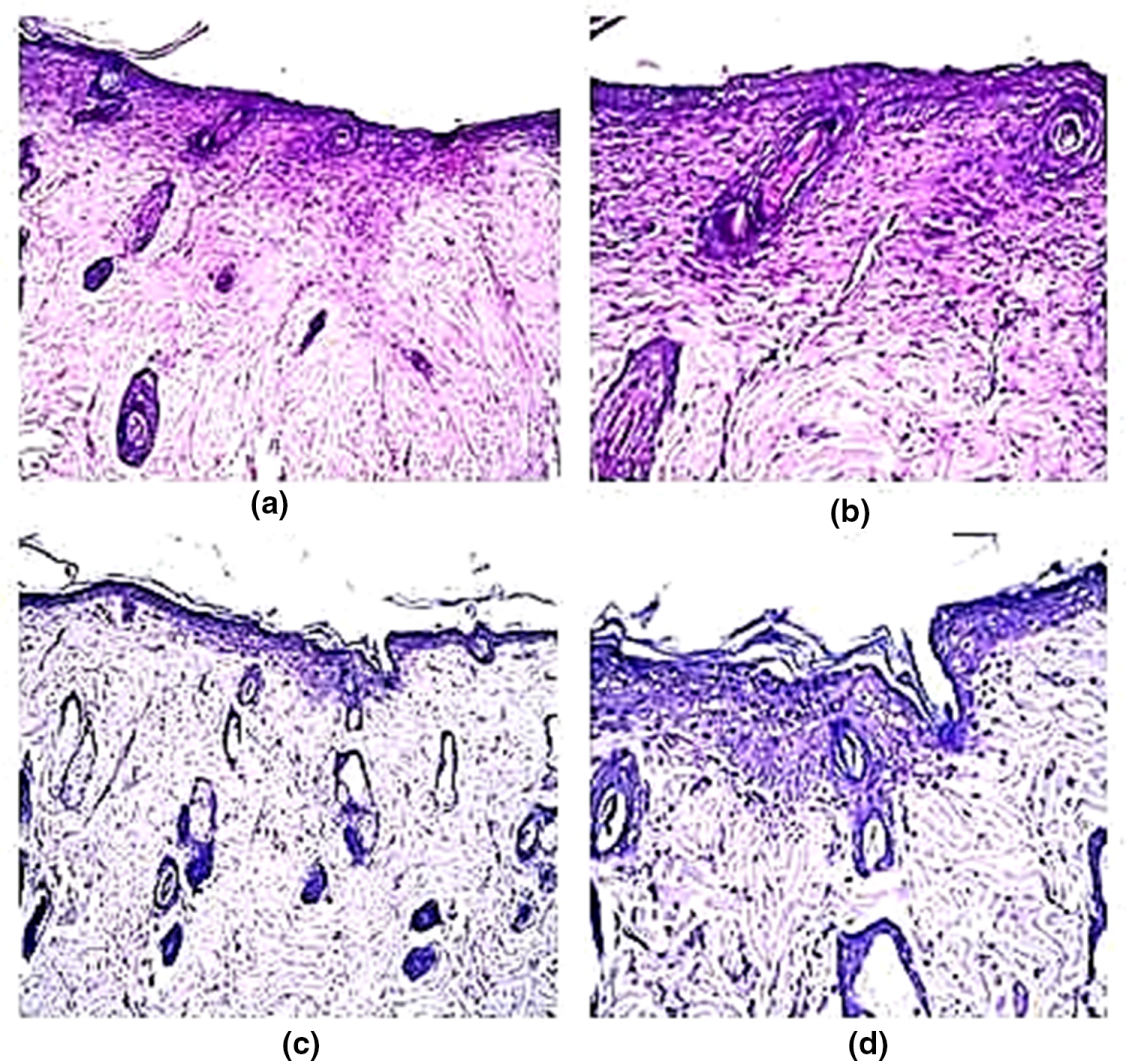
**a** Showing focal area of regeneration in epidermis with underlying area of granulation tissue formation (fibrosis and newly formed capillaries) in the underlying dermis. **b** Showing the magnification of a to identify the regenerated epidermis and granulation tissue in underlying dermis. (H&E, × 40). **c** Showing normal histological structure of the epidermis and underlying dermis with hair follicles and sebaceous glands (H&E, × 16). **d** Showing the magnification of **c** to identify the normal histological structure (H&E, × 40)

In group (T), there was a focal area of regeneration in the epidermis associated with granulation tissue formation (fibrosis with newly formed capillaries) in the underlying dermis (Fig. 10a, b). In some mice of the same group, there was no histopathological alteration in the epidermis as well as the underlying dermis with hair follicles and sebaceous glands (Fig. 10c, d).

## Discussion

Selection methods for probiotics usually encompass checking for probiotic properties such as heat, acid, bile salt and NaCl tolerance. Also, probiotics are examined for their antimicrobial activity to study their capability to fight pathogens in the GI system. In addition, the generation of EPSs has been considered a valuable feature that promotes the probiotic settlement in the gut, as well as their potential to suppress the detrimental enzymes of intestinal microflora (Sornplang and Piyadeatsoontorn 2016). So, the RO30 strain isolated from Romi cheese was chosen due to its capability to biosynthesis high quantity of EPS and its good probiotic features. LAB strain were previously isolated from different sources such as spontaneously fermented pickles (Mıdık et al. 2020), milk sources (Imran et al. 2016) and kefir grains (Wang et al. 2018).

Certain *lactobacilli* are known to produce EPS. The generated amount of EPS differed between bacterial strains revealing that EPS output is influenced not only by the strains employed but also by the nutritional and non-nutritional circumstances (Khalil et al. 2018). Imran et al. (2016), Khalil et al. (2018), Aarti and Khusro (2019), Mıdık et al. (2020) and Rajoka et al. (2022) estimated 250 mg/l, 750 mg/l, 450 mg/l, 515 mg/l, and 590 mg/l, of EPS output, respectively from *Lactobacillus* sp. in the existence of sucrose reinforced media.

Growth temperature is an important physical circumstance that regulates the growth and metabolism of microbes. *L. plantarum* RO30 grew well at 25, 35 and 40 °C, but a reduction in growth was detected at 45 °C. In this concern, Mulaw et al. (2019) isolated LAB that were able to grow at both 15 °C and 45 °C except E031 was not found to grow at 45 °C.

Probiotic bacteria tolerance to different pH and salt concentrations which can be a common occurrence during food processing is another essential merit of probiotics as it gives the opportunity to the bacteria to start metabolism, which generates acids, that further suppresses the growth of unwanted organisms. *L. plantarum* RO30 has a promising possibility for use in the food industry, due to its ability to tolerate a variety of growth-inhibiting surroundings including high salt concentrations (4.5 and 6.5) and wide pH ranges (4.5, 5.5, 6.5, 7.5 and pH 8.5). In concurrence with our results, Jawan et al. (2021) illustrated that the *Lactococcus lactis* Gh1 strain was tolerant to high salt concentrations (0.1 to 4%). Prabhurajeshwar and Chandrakanth (2019) also informed that dissimilar *Lactobacillus* isolates from commercial yoghurt were found to be salt-tolerant at concentrations ranging from 1 to 6%. Contrary to these findings, Mulaw et al. (2019) stated that LAB isolate (E052) from traditionally fermented Ethiopian food products was unable to grow at 6.5% salt concentrations. It is henceforth proposed that the tolerance of probiotic bacteria to various grades of salt concentrations and other growth-inhibiting circumstances may be species and strain-dependent.

The viability and existence of probiotic bacteria in acidic circumstances is one of the urgent criteria for providing therapeutic functions. The pH of the stomach generally ranges from pH 2.5 to pH 3.5 and this is a barrier against the entry of bacteria into the intestinal tract (Boke et al. 2010). In concurrence with our findings, Cebeci and Gürakan (2003), Begley et al. (2006), Tulini et al. (2013) and Talib et al. (2019) also reported good survival rates for *Lactiplantibacilli* after 90 min in MRS broth at pH 3.0 and 3.5.

The ability of *L. plantarum* RO30 to survive in bile salts assists colonization and metabolic activity of bacteria in the host’s small intestine (Nawaz et al. 2017; Saif and Sakr 2020). In vivo, bile salts act as biological detergents that emulsify and solubilize lipids, thereby playing an essential role in fat digestion (Begley et al. 2006).

Characterization of the bacterial cell surface is one of the in vitro tests, which were investigated to provide information about the probiotic nature and it varies substantially among probiotic *Lactiplantibacillus* strains (Vinderola and Reinheimer 2003). Hydrophobicity of some known cultures such as *L. plantarum* ATCC8014, *L. pentosus,* ATCC8041, *L. casei* NCIMB 3254, and *L. delbrueckii* NCIM 2025 was found to be 5.5, 6.5, 6.2 and 3.7%, respectively, and all are very less than *L. plantarum* RO30. The lessened hydrophobicity of some of the isolates could be attributed to the reason for EPS production by these strains. The existence of EPS fractions will enhance a significant reduction in probiotic strains adherence (Ruas-Madiedo et al. 2002). EPS could stick to mucus directly and then compete with probiotics for adherence. Furthermore, the difference in cell surface hydrophobicity could be attributed to species-specific differences in the density of cell surface protein expression (dos Santos et al. 2021). Although the hydrophobicity of a bacterial surface can alter its adhesion capacity to diverse surfaces, it is not a provision for vigorous attachment to human gastrointestinal cells (de Souza et al. 2019).

Antimicrobial activity is a critical aspect of probiotic performance. Some compounds such as metabolites, organic acids, hydrogen peroxides, diacetyl, and bacteriocins produced during LAB growth may be responsible for LAB strains antimicrobial activity (Zuo et al. 2016).

Hemolysis is a prominent virulence factor among pathogenic microorganisms. Thus, in this regard, the tested RO30 strain was γ-hemolytic and cannot discharge cellular toxins such as cytolysin or streptolysin which are hazardous to the immune system because they enhance virulence in animal models (Motey et al. 2021). So, the *L. plantarum* RO30 strain manifested no pathogenicity traits and could be considered safe. This finding was resemble a preceding study that revealed *Lactiplantibacillus* spp. possessed no hemolytic activity (Talib et al. 2019).

It is urgent to study the antibiotic resistance of the isolated *Lactiplantibacillus* spp. to avoid severe medical dangers. Probiotic bacteria can possess intrinsic and mobile genetic elements that ascertain resistance to a wide variety of antibiotic classes which could transfer to pathogens. Also, the lack of an antibiotic's target site in a LAB cell may explain the resistance to that antibiotic (Abushelaibi et al. 2017). Georgieva et al. (2015); Talib et al. (2019); Adebayo-Tayo and Fashogbon (2020) reported that *Lactiplantibacillus* spp. are generally sensitive to β-lactams antibiotics, such as penicillin, and broad-spectrum antibiotics like tetracycline.

EPS biosynthesis by strain *L. plantarum* RO30 isolated from Romi cheese on 5% sucrose enclosing MRS adjusted medium was studied. The chemical composition analysis ascertained that the REPS sample possessed a considerable amount of carbohydrates. The former studies have found that LAB-EPS has high carbohydrate content (Li et al. 2014; Wang et al. 2015; Imran et al. 2016; Zaghloul and Ibrahim 2022).

The results of the REPS monosaccharide composition strongly indicated that the REPS is a heteropolysaccharide with glucuronic acid, mannose, glucose, and arabinose as monomer units with a molar ratio of 2.19:0.1:0.536:0.104, respectively. The most common monosaccharides present in the LAB EPS are galactose, mannose, glucose, fructose, rhamnose, arabinose, and xylose. Furthermore, sugar derivatives such as *N*-acetyl glucosamine and *N*-acetyl galactosamine are found (Rajoka et al. 2019). Tang et al. (2015) informed that *L. plantarum* EPS consisted of ribose, rhamnose, arabinose, xylose, mannose, glucose, and galactose in a molar ratio of 2:1:1:10:4:205:215, respectively. Moreover, Liu et al. (2017) proved that the EPS from *L. Plantarum* WLPL04 from human breast milk is made up of xylose, glucose, and galactose in a ratio of 3.4: 1.8: 1.0, respectively. The kind of strains, cultural conditions and media constitution affect the monosaccharide composition of EPS.

Mw of REPS was similar to that (4.4 × 10^4^ Da) of the EPS of* L. plantarum *EP56 (Tallon et al. 2003) but lower than the EPS (1.1 × 10^5^ Da) from *L. plantarum* YW11 (Wang et al. 2015) and the EPS (1.15 × 10^6^ Da) of *L. plantarum* C88 (Zhang et al. 2013), but higher than that (1.83 × 10^4^ and 1.33 × 10^4^ Da) of the EPS of *L. plantarum* BC-25 (Zhou et al. 2016) and the (EPS-ETOH) (3.35 ± 2.89 × 10^4^ Da) from *L. plantarum* C7 (Ziadi et al. 2018). The polydispersity index (PI, Mw/Mn), which reflects the heterogeneity degree of the polymer’s chain lengths, was 1.53 (> 1) indicating a heterogeneous population in terms of polysaccharide chains size. Similar results of PI were reported for AEPS from *L. acidophilus* (1.54 ± 0.09) (Yang et al. 2021), and EPS from *L. plantarum* AR307 (1.57) (Feng et al. 2021) revealing a narrow molecular weight distribution.

Fourier transform infrared spectroscopy is a vibrational spectroscopic technique that is used for the identification and structural analysis of the functional groups in EPS (Hu et al. 2021). Comparing the FT-IR spectrum of the functional groups present in REPS to the FT-IR spectra analysis of the other polysaccharides discussed in the preceding publications confirmed that the REPS is a carbohydrate in nature. The polysaccharide exhibited a broad absorption peak around 3400 cm^−1^ characteristics of massive amounts of OH groups proving the polysaccharide nature of the compound (Ziadi et al. 2018). The C–H stretching vibration of the sugar ring was observed at 2934 cm^−1^, while its bending vibration appeared at 1558 cm^−1^ (Min et al. 2019; Rajoka et al. 2019). An intense extending peak at around 1653 cm^−1^ was attributed to the stretching vibration of the carbonyl group (C=O) and a carboxyl group. A weak peak at around 1558 cm^−1^ corresponds to the bending vibration of the N–H bond of peptides in proteins (Liu et al. 2017). Also, the small peaks at 1454, 1418, and 1235 cm^−1^ which were observed in the sample, might prove the presence of symmetric stretching of carboxyl group COO^−^. The strong peak at around 1073 cm^−1^ was attributed to the vibration of the glycosidic bond C–O–C of glucose (Ziadi et al. 2018). A signal at 784 was attributed to α-glycosidic linkage. Moreover, a peak at 880 cm^–1^ assigned for β-glycosidic linkage was also detected. Thus, REPS has both α and β-glycosidic linkage. Our results were in agreement with former results about functional groups of *Lactiplantibacilli* EPS informed by Wang et al. (2015), Amiri et al. (2019) and Rajoka et al. (2019).

In addition to FT-IR spectroscopy, ^1^HNMR spectroscopy is an essential tool to provide valuable information about the structure of a polysaccharide. Conventionally, the ^1^HNMR spectrum of polysaccharides consists of three regions; the anomeric region (*δ* 4.5–5.5 ppm) demonstrates the anomeric signal of sugar residues, ring proton region (*δ* 3.1–4.5 ppm) reveals the proton linkage to C2–C6, and alkyl region (*δ* 1.2–2.3 ppm) (Hu et al. 2021). For REPS, the signals at chemical shift 4.99–5.89 ppm were due to α-anomeric proton, while the peaks around *δ* 4.38 belonged to β-anomeric configuration. The signals around *δ* 3.38–4.09 ppm indicate the complexity of the structure as well as the proton of the *N*-acetyl group of REPS residues (Dong et al. 2018). The ^1^HNMR spectrum confirmed the presence of exopolysaccharide and was in assent with that of FT-IR spectra analysis. Similar results were obtained by Rajoka et al. (2022).

The in vitro determination of the antioxidant activities increased with an increase in REPS concentrations. REPS radical scavenging activity might be attributed to the existence of hydroxyl groups and other functional groups in the REPS, which can give electrons to lessen the radicals to a more stable form or react with the free radicals to end the radical chain reaction (Shen et al. 2013). Dilna et al. (2015) declared that EPS extracted from *L. plantarum* RJF4 at a concentration of 2 mg/ml manifested scavenging activity of 23.63%. While, in vitro, antioxidant activities of the EPS-YO175 and EPS-OF101 from *L. plantarum* YO175 and OF101 were 56.9% and 51.3%, respectively at 4.0 mg concentration (Adesulu-Dahunsi et al. 2018).

Fe (III) ion reduction is frequently used as an indicator of electron-donating activity, which is highly linked with other antioxidant properties. Trabelsi et al. (2017) informed that the chemical composition of polysaccharides, especially the reductive nature of their constitutive monosaccharides may positively influence their reductive power. Therefore these findings confirm the reduction potential power of the REPS, which was revealed to be composed of mannose, glucose, and arabinose. Liu et al. (2011) registered a reducing capacity of 0.75 and 0.73 at 10 mg/ml for 101EP and 102EP, respectively**.** A reducing capacity of 0.41 and 0.34 was reported for EPS-YO175 and EPS-OF101, respectively at 4 mg concentration (Adesulu-Dahunsi et al. 2018).

Measuring the iron-ferrozine complex formation was utilized to determine the Fe^+2^ chelating ability of REPS. One of the important antioxidant mechanisms is metal ion chelating activity, which reduces the number of transition metals that catalyze lipid peroxidation. Metal ion chelating agents with a structure containing OH, –COOH, C=O, and –O– in a required structure–function position are assumed excellent secondary antioxidants since they can construct bonds with metals, reducing the redox potential and thus stabilize the iron ion oxidized form (Li et al. 2014). According to our results, REPS has a similar structure and this may be a possible explanation for its strong chelating activity. An aforementioned study by Li et al. (2014) reported metal ion chelating activity up to 39%, 46%, 53%, and 59% and at a concentration of 4 mg/ml for crude EPS-3, EPS-2, EPS-1, and EPS extracted from *L. helveticus* MB2-1, respectively. Also, 102EP and 101EP were obtained from *L. plantarum* NTU 102*,* and *L. paracasei* subsp. *Paracasei* NTU 101 in a preceding study by Liu et al. (2011), possessed 29% and 54% chelating power, respectively, at a concentration of 10 mg/ml. REPS employed by our study had higher metal ion chelating activity than what has been reported for EPS, 101EP, and 102EP.

The improvement of the wound healing process has been linked to a number of factors. The therapeutic and protective effects of antioxidant molecules on wound healing have been described by Süntar et al. (2012). They counteract the excess of reactive oxygen species (ROS) and proteases commonly produced by neutrophil buildup in the wound area, in addition to eliminating inflammatory products. Because an excess of ROS can cause serious consequences such as fibroblast cell death, decreased skin lipid elasticity, and oxidative damage to protease inhibitors, antioxidants can help to prevent these negative outcomes. So, REPS antioxidant properties may be one of the most effective contributors to better burn wound healing. Our finding suggested that topical application of cream base containing 0.5% REPS increased regularly the wound contraction percentage. In opposite, the normal saline-treated burn wounds healed much more slowly. According to Maalej et al. (2014), the addition of EPS22 produced by *Pseudomonas stutzeri* AS22 gel to cutaneous full thickness excision wounds in a rat model every two days improved wound healing activity considerably and resulted in 100% closure after 12 days of wound induction. Furthermore, histological examination of samples revealed extensive tissue regeneration, as evidenced by the occurrence of well-organized derma and epidermis stratum.

## Conclusion

In the present study, *L. plantarum* RO30 isolated from Romi cheese was found to have potentially probiotic characteristics. It is suggested that this strain can be a promising candidate for exploitation in the food industry as a prospective probiotic culture with human health benefits. Also, a biologically active heteropolysaccharide named REPS was extracted from *L. plantarum* RO30. REPS was explored for its potential use as a burn wound healing agent. The results of in vitro antioxidant activities and the in vivo wound healing performance provided support for the promising use of REPS for applications as a therapeutic agent for burn wound healing.
